# Differences in Physical Activity Patterns among Korean Adolescents during and after COVID-19

**DOI:** 10.3390/healthcare11111611

**Published:** 2023-05-31

**Authors:** Juseok Yun, Seungman Lee

**Affiliations:** 1 Department of Physical Education, Korea University, Seoul 02841, Republic of Korea; tennis2667@korea.ac.kr; 2 Department of Sports Science, Hankyong National University, Gyeonggi 17579, Republic of Korea

**Keywords:** adolescent health, coronavirus, pandemic, physical activity, moderate to vigorous physical activity school, Republic of Korea

## Abstract

Due to the COVID-19-induced social distancing restrictions, adolescents’ physical activity declined and their level of health and fitness decreased. In March 2023, the Korean government established the beginning of the post-COVID-19 era by declaring that indoor masks were now “recommended” rather than “mandatory”. Consequently, adolescents, whose physical activity decreased during COVID-19, began to participate in such activities again. This study aimed to verify the differences in adolescent physical activity during COVID-19 and after COVID-19. To achieve the study’s purpose, an online survey was conducted twice, using the International Physical Activity Questionnaire, for 1143 Korean adolescents in 2022 and 2023. The following results were derived through frequency analysis, descriptive statistical analysis, and an independent variables *t*-test. First, moderate-to-vigorous physical activity was higher during the post-COVID-19 period than during COVID-19 (*p* = 0.018). Second, high-intensity (*p* = 0.018), moderate-intensity (*p* = 0.030), and low-intensity (*p* = 0.002) physical activities and total leisure-time physical activities (*p* = 0.003) were all higher during the post-COVID-19 period than during COVID-19. Third, high-intensity (*p* = 0.005), moderate-intensity (*p* = 0.003), low-intensity (*p* = 0.003) activities and total physical activities in school (*p* = 0.001) were all higher during the post-COVID-19 period than during COVID-19. Fourth, there was no difference in the commuting times for cycling (*p* = 0.515) and walking (*p* = 0.484) and the total physical activities during commuting (*p* = 0.375) during and after COVID-19. Based on these results, the methods to help adolescents form correct habits for leading a healthy life are discussed.

## 1. Introduction

The definition of physical activity is expanding with time. Caspersen et al. [1] initially defined physical activity as “Any bodily movement produced by skeletal muscles that results in energy expenditure”. While this definition provides a specific physical activity, it is a narrow concept that focuses on specific mechanistic acts involving skeletal muscles and energy intake [2]. The US National Institutes of Health [3] introduced the notion of “health benefits” and defined physical activity as “bodily movement produced by skeletal muscles that requires energy expenditure and yields health benefits”. However, since certain physical activities such as overtraining, repetitive strain, and physical combat may not necessarily be beneficial to health, it is necessary to address the criticism that physical activity does not universally benefit everyone’s health [2]. Consequently, Piggin [4] supplemented and expanded the existing concept of physical activity by defining it as “Physical activity involves people moving, acting, and performing within culturally specific spaces and contexts, influenced by a unique array of interests, emotions, ideas, instructions, and relationships”. In essence, the definition of physical activity has evolved to encompass multiple dimensions with the changing times. The World Health Organization (WHO) [5] defines physical activity as “Any bodily movement produced by skeletal muscles that requires energy expenditure” and provides recommendations for the amount of physical activity to be performed by children, adolescents, adults, and older adults. In this study, the amount of physical activity was assessed using the widely used International Physical Activity Questionnaire (IPAQ), based on the WHO’s definition of physical activity.

Despite the difficulty in defining physical activity, physical activity is one of the predictors of a healthy life as it has a positive effect on the prevention of various conditions, such as heart disease, hypertension, diabetes, depression, and obesity [6,7,8]. Therefore, it should be promoted and encouraged throughout one’s lifecycle, including in childhood, adolescence, adulthood, and old age [9,10]. In particular, physical activity during adolescence affects not only the aspects of physical health, such as the maintenance of normal weight, reduction in weight gain, and prevention of obesity, but also mental health and academic achievement [11]. In addition, continuous and rigorous physical activity in adolescence leads to physical activity in adulthood and has a positive effect on public health; therefore, continuous attention needs to be given to this subject [12,13].

In 2020, the World Health Organization (WHO) recognized the importance of physical activity in adolescence, recommending at least 60 min of moderate-to-vigorous physical activity (MVPA) every day to maintain and improve adolescent health [5]. However, more than 80% of adolescents worldwide do not meet the recommended level, and it is reported that physical activity tends to decrease as adolescents move to higher grades, raising apprehensions regarding adolescent health [14]. To elaborate, overseas reports showed that the fulfillment rates of the recommended physical activity criteria for 9- and 15-year-olds were 96.7% and 62%, respectively [15]. By grade, 87% of grades 4–6 (elementary school), 54.2% of grades 7–9 (middle school), and 25.1% of grades 10–12 (high school) reported a red alert for adolescent health [16]. In Korea, 64% of male and 42% of female students in grades 5–6, 49% of male and 26% of female students in grades 7–9, and 7% of female students in grades 10–12 met the recommended standards. In other words, Korean adolescents do not meet the level of physical activity recommended by the WHO, and the amount of physical activity tends to decrease rapidly as they move to higher academic grades. In addition, the rate of satisfaction with the recommended standards for physical activity in Korea was significantly lower than that of other countries [17,18].

On 11 March 2020, the WHO declared the coronavirus infection as a global pandemic. Society as a whole has undergone unprecedented changes in many areas, such as those related to the economy, education, and personal lifestyles, due to the unexpected appearance of the coronavirus disease 2019 (COVID-19) [19]. As the COVID-19 death toll increased exponentially, governments across the world implemented policies. These included hand washing, mask wearing, self-isolation, social distancing, and national lockdown to prevent the spread of the virus [20]. Although these policies have been effective in preventing COVID-19 and suppressing its spread, they have taken a toll on personal lifestyles. Our daily lifestyles changed due to social distancing; “increased sedentary activity time, decreased physical activity (less active, more sedentary)” were the most noticeable changes [21,22]. Moreover, there has been a rapid increase in obesity in the population, along with the decreased physical activity and rising mental health concerns pertaining to life satisfaction and happiness [23,24,25].

School, which is generally the main area of adolescent physical activity, was partially closed because of quarantine measures during the pandemic. As school classes were converted to non-face-to-face online classes, adolescent physical activity decreased. Looking at the amount of reported adolescent physical activity during the COVID-19 period, the physical activity of European adolescents decreased from 30 to 15 min [26], and the MVPA fulfillment rate among American adolescents decreased from 16.1% to 8.9% [27]. In China, physical activity time per week significantly decreased from 540 to 105 min [19]; among Korean adolescents, MVPA time decreased by 9 min, on average, and the fulfillment rate decreased by 7% [28,29]. Overall, COVID-19 exacerbated the problem of the adolescent sedentary lifestyle that is continuously rising. To correct this, more attention needs to be paid to this problem of adolescent physical activity.

On 20 March 2023, expectations for an announcement of the official end of the pandemic were growing in Korea as mandatory mask wearing was lifted on public transportation and in pharmacies. During COVID-19, society faced a significant social shift through changes in everyday routines [30]. Today, people want to return to pre-COVID-19 routines [31]. In particular, as the impact of decreasing healthcare and physical activity was felt acutely during the COVID-19 period, efforts to restore it to previous levels after COVID-19 have gained importance [32]. Thus, to assess this effectively, we need to compare the physical activity levels of adolescents during COVID-19 with those of the post-COVID-19 era and to seek alternatives for adolescent healthcare and physical activity problems in the current post-COVID-19 era. Unfortunately, although many studies have compared pre- and post-COVID-19 physical activity [22,26,27], few have compared the physical activity of adolescents during COVID-19 with that of the post-COVID-19 era.

To fill this literature gap, our study compared the characteristics and levels of the physical activity of Korean adolescents using the IPAQ, during COVID-19 and after it. In the past, the physical activity of adolescents was measured through subjective methods, such as questionnaires, and objective methods, such as the three-dimensional accelerometer, which is highly valid and reliable in measuring physical activity [33,34]. However, using it during the COVID-19 pandemic raised concerns about spreading the disease. Moreover, its usage limits the size of the data samples. In contrast, the IPAQ, developed by the WHO, is easy to measure and compare worldwide. Additionally, it has been validated through a comparison with physical activity directly measured using the accelerometer [35]. Consequently, it is being applied globally.

In the past three years, Korean society has faced the challenges posed by the COVID-19 pandemic, which has led to a renewed recognition of the importance of physical activity. Given the current circumstances, this study aims to underscore the significance of physical activity for adolescents. Physical activity plays a crucial role in the development of adolescent health [17,18], and numerous studies [6,7,8,11,12,13,14,15,19] have consistently highlighted its importance. What these studies commonly emphasize is that engaging in vigorous physical activity during this life stage establishes the foundation for lifelong health, and that physical activity is essential to the leading of a healthy life. Building upon the findings of previous studies [6,7,8,11,12,13,14,15,17,18,19], this study seeks to address the challenges related to physical activity in the field of physical education, which is presently concerned with the health issues that young people are facing in the aftermath of the pandemic.

By utilizing the IPAQ, this study aims to gather essential information that will help to provide solutions in the context of improving adolescent physical activity and to address the emerging health problems resulting from the pandemic. The research hypotheses for this study are as follows: during the post-COVID-19 period, MVPA levels are higher than those observed during the COVID-19 period in three primary contexts: leisure time, school, and commuting.

## 2. Materials and Methods

### 2.1. Design and Participants

In this study, a cross-sectional design was used. The study population was Korean adolescents. To select a representative sample, convenience sampling was used; 1143 adolescents (570 during COVID-19 and 573 after COVID-19) in middle and high school were selected. The estimate of the sampling error was found to be ±1.85%p. As Korean adolescents are a homogeneous group, it was judged that the sample selected in this study sufficiently represented the characteristics of the target population.

The survey was conducted twice (in July 2022 during COVID-19 and in April 2023 after COVID-19); there was no overlapping of participants in the two surveys. The average age of the participants was 15.95 years (standard deviation, SD = 1.117). The questions on economic level and academic performance were answered according to the subjective criteria considered by the respondents. The Physical Activity Promoting System (PAPS) classifies all elementary, middle, and high school students in Korea into five levels by measuring five physical strengths (power, cardiorespiratory endurance, muscular strength and endurance, flexibility, and body composition). The obesity grade was classified into underweight, normal, mildly obese, and obese, using the body mass index (BMI) method.

The general characteristics of the participants are presented in Table 1. Specifically, there were 577 boys and 566 girls; according to the PAPS, 289 adolescents were classified into the 1st grade, 334 in the 2nd grade, 378 in the 3rd grade, 97 in the 4th grade, and 45 in the 5th grade. Finally, as per the obesity grade, 424 adolescents were underweight, 524 normal, 147 mildly obese, and 48 obese.

### 2.2. Instruments and Variables

This study used the IPAQ to measure the physical activity of the participants. As a self-administered physical activity questionnaire used widely [37], its questions investigate all physical activities, including those performed while doing household chores, leisure-time activities, indoor activities, outdoor activities, work-related activities, and transportation activities. Furthermore, it captures information such as the amount of walking, moderate physical activity, and vigorous physical activity over the previous seven days. The IPAQ has been adapted in 21 languages, and its reliability and validity have been verified in many studies [17,35]. This study used the Korean IPAQ long version; its reliability and validity have been confirmed in studies targeting Korean adolescents [17,38].

This study asked the following question: “During the past seven days, how many days did you engage in vigorous physical activity for at least 10 min at school”? The respondents answered with the number of days of being either physical active or inactive. Additionally, the survey asked, “How much vigorous physical activity do you do at a time”? The response to physical activity lasting more than 10 min comprised three areas: leisure-time physical activities, physical activities in school, and physical activities during their school commute. The respondents were asked to indicate how many hours per day and how many days per week they engaged in physical activity, according to the intensity of the physical activity they participated in by activity area. In general, a metabolic equivalent (MET) of less than 3 is considered low-intensity physical activity; 3 to less than 6 is considered moderate-intensity physical activity; and 6 or more is considered high-intensity physical activity [39]. As there may be differences in the perceived intensity of physical activity among the respondents, this study included examples in each area in the questionnaire.

Next, the general characteristics of the research participants were classified into five categories: sex, household economic level, academic performance, PAPS grades, and obesity level. Specifically, obesity was calculated using the BMI formula. Data collection was conducted through a self-report online questionnaire (Google Forms) according to the purpose of the study.

### 2.3. Ethical Statement

The procedures of this study were as per the ethical standards of the responsible committee on human experimentation (institutional and national) and the Helsinki Declaration of 1975, as revised in 2013. This study was registered and approved by the Ethics Committee of the Korea National University of Education (registration no-KNUE-202305-SB-0060-01).

### 2.4. Data Analysis

This study used SPSS for Windows (version 18.0; IBM Corp., Armonk, NY, USA) to analyze the collected data. This study examined the following: (1) frequency, to determine the participants’ demographic characteristics. (2) Next, the Kolmogorov–Smirnov test was applied, considering that the variables followed a normal distribution; (3) Cronbach’s alpha was used to verify the reliability of the physical activity scale; (4) the number of cases, minimum value, maximum value, average, and standard deviation calculations were determined to confirm the amount of physical activity of the adolescents; and (5) an independent t-test was conducted to verify the difference in physical activity in adolescents during and after COVID-19, with the statistical significance level set at 0.05. At this point, as suggested by Sanz-Martín et al. [40], Cohen’s d statistic and the 95% confidence interval of the difference between the groups were confirmed. This study investigated the economic level of families and the level of academic achievement. By employing a three-way analysis of variance (ANOVA), it was anticipated that more diverse results could be obtained by comparing the pre- and post-pandemic periods. However, Natekar et al. [41] caution that utilizing a three-way ANOVA might complicate the interpretation of the results. Given the specific focus of this study on comparing adolescent physical activity during and after COVID-19, it was decided not to include additional factors such as the economic level of families, level of academic achievement, and measurement period (during and after COVID-19). This approach was taken to avoid potential errors that could have detracted from the study’s intended purpose.

## 3. Results

### 3.1. Verifying the Reliability and Validity of the Scale

As this study was conducted as an online survey, there were no responses with missing data. No participants were excluded from the study. This study used the Delphi technique to verify the content validity of the IPAQ. The IPAQ was previously translated into Korean and verified as reliable and valid in various studies [17,18]; however, as it was previously verified before the COVID-19 pandemic, the content validity was verified in this study. In the Delphi technique, a closed-type questionnaire with a two-point scale based on previous research is used for targeting five experts (two professors majoring in physical education and three PhD graduates). This study found that all items had content validity that exceeded 50% of the total score. Next, this study verified the internal consistency and reliability of the questionnaire. If the α coefficient is 0.7, it can be said to be reliable [42]. The reliability of the IPAQ in this study ranged from 0.730 to 0.845, by area (Table 2).

### 3.2. Differences in Moderate-to-Vigorous Physical Activity during and after COVID-19

In this study, MVPA was calculated by adding leisure physical activity to school physical activity. As shown in Table 3, MVPA was significantly higher after COVID-19 (M = 450.73) than during it (M = 337.78) (t = −2.360, *p* = 0.018).

### 3.3. Differences in Leisure-Time Physical Activities during and after COVID-19

As shown in Table 4, high-intensity leisure-time physical activity was significantly higher after COVID-19 (M = 221.51) than during COVID-19 (M = 161.42) (t = −2.363, *p* = 0.018); moderate-intensity leisure-time physical activity was significantly higher after COVID-19 (M = 229.23) than during COVID-19 (M = 176.36) (t = −2.167, *p* = 0.030); low-intensity leisure-time physical activity was significantly higher after COVID-19 (M = 395.42) than during COVID-19 (M = 326.67) (t = −3.064, *p* = 0.002); and total leisure-time physical activity was significantly higher after COVID-19 (M = 846.15) than during COVID-19 (M = 664.45) (t = −2.936, *p* = 0.003).

### 3.4. Differences in Physical Activities in School during and after COVID-19

As shown in Table 5, high-intensity physical activities in schools were significantly higher after COVID-19 (M = 85.45) than during it (M = 53.55) (t = −2.797, *p* = 0.005). Moderate-intensity physical activity in schools was significantly higher after COVID-19 (M = 80.31) than during it (M = 49.89) (t = −2.939, *p* = 0.003); low-intensity physical activities in school were statistically significantly higher after COVID-19 (M = 97.00) than during it (M = 73.70) (t = −2.988, *p* = 0.003). Similarly, total physical activity in school was significantly higher after COVID-19 (M = 262.76) than during COVID-19 (M = 177.14) (t = −3.466, *p* = 0.001).

### 3.5. Differences in Physical Activities during Students’ Commute Amid and After COVID-19

As shown in Table 6, there was no statistically significant difference in bicycle commuting during (M = 22.04) and after COVID-19 (M = 25.14) (t = −0.651, *p* = 0.235); walking commuting time was not significantly different during (M = 134.29) and after COVID-19 (M = 141.05) (t = −0.700, *p* = 0.350). Thus, there was no statistically significant difference in total physical activity for commute time during (M = 156.32) and after COVID-19 (M = 166.18) (t = −0.888, *p* = 0.186).

## 4. Discussion

This study aimed to examine differences in the MVPA of Korean adolescents during and after the COVID-19 pandemic. An online survey of 1142 adolescents was conducted in 2022 and 2023 via Google Forms to assess and compare the physical activities of Korean adolescents during and after COVID-19.

### 4.1. Interpretation of the Findings

From a positive perspective, this study’s hypotheses were confirmed, meaning that physical activity among adolescents generally increased after COVID-19. First, adolescent MVPA was higher after COVID-19 than during it. The importance of high-intensity physical activity during COVID-19 has been emphasized in several studies [43,44,45]. The increase in MVPA in physical activities during leisure-time, school, and commuting can be interpreted as follows. The social atmosphere made it difficult for adolescents to freely participate in various forms of high-intensity physical activity because of the government’s social distancing measures during COVID-19. However, after COVID-19, as restrictions were lifted, an environment where people could actively participate in physical activities in daily life was created. In addition, COVID-19 increased the interest of many people in improving their health and immunity. As more people recognized the role of MVPA in this process, many became interested in MVPA. As MVPA has various physical effects, such as the relieving of stress, an increasing number of adolescents want to improve their health and immunity through it. Moreover, various educational programs in schools focused on maintaining proper health during COVID-19 have continued after COVID-19. Based on the current results, it is necessary to emphasize the importance of physical activity for adolescents. For this reason, it is necessary to guide physical activity so that it becomes a habit.

Second, the adolescents’ high-, moderate-, and low-intensity physical activities and total leisure-time physical activities were higher after COVID-19 than during COVID-19. As it is still early in the post-COVID-19 period, no comparative study on COVID-19 and post-COVID-19 physical activity has yet been reported. Although people’s lives during the COVID-19 and post-COVID-19 periods differ greatly, in this study it can be assumed that the leisure-time physical activity of adolescents increased after COVID-19. During the pandemic, many schools switched to online or blended classes that combined face-to-face and online instruction. As students stayed at home to minimize contact with others, the amount of their leisure-time physical activity decreased [46,47]. However, in the post-COVID-19 era, people are again enjoying cycling, walking, mountain climbing, and sports or outdoor activities. Therefore, it is likely that this change spread to adolescents and increased their leisure-time physical activity. To increase adolescents’ physical activity in their spare time, schools should guide them to take an interest in sports. For example, it is possible to measure the amount of physical activity by using smart devices such as smart watches. Furthermore, smartphone applications can be used to compete with friends or to evaluate physical education classes.

Third, adolescent high-, moderate-, and low-intensity activities and total physical activities in the post-COVID-19 period were higher in schools than during COVID-19. Previous studies [22,26,27,28,29] confirmed that the majority of adolescents participated in physical education classes and physical activities in school before the pandemic, with the proportion decreasing after the pandemic. Furthermore, a study by Rossi et al. [45] reported that participation in physical activities in schools decreased as the use of school playgrounds and gymnasiums was restricted during the pandemic. Thus, the pandemic reduced adolescent physical activity in schools. However, after COVID-19, many schools and businesses have gone back to face-to-face interactions; adolescents have returned to school, and parents have returned to work. Consequently, it can be assumed that physical activity in the schools increased as the time spent at school increased. During COVID-19, school physical activity was limited to specific periods of physical education for adolescents; however, after COVID-19 adolescents are able to engage in various physical activities, taking advantage of not only physical education for such activities, but also breaks and lunchtime. Additionally, physical activity spaces in schools had been closed because of social distancing policies. However, as we enter the post-COVID-19 era, spaces for adolescents to engage in physical activities have re-opened, providing environments to participate freely in physical activities in various areas within their schools. In response to COVID-19, some schools have remodeled their gymnasiums to include more outdoor exercise facilities and have improved and expanded sports facilities for more exercise indoors. These changes are providing opportunities for adolescents to enjoy exercise with increased opportunities for physical activity in the post-COVID-19 period. To increase adolescents’ physical activities in school, school sports clubs should be further activated, and sports facilities in schools should be expanded to enable them to participate in the desired sports anytime and anywhere.

Fourth, concerning the adolescents’ cycling or walking or total physical commuting activity time, there were no differences during and after COVID-19. Zainafree et al. [46] reported that the proportion of adolescents who traveled by bicycle or walked increased as the use of public transportation decreased. Cho et al. [47] indicated that most adolescents commuted to and from school by bicycle or walking during the pandemic. However, during COVID-19, due to concerns about infection, these adolescents walked or biked relatively short distances rather than use public transportation. Simultaneously, to prevent a decrease in the physical activity of adolescents during COVID-19, education to increase physical activity at school was planned. Accordingly, adolescents also tried to remain physically active to maintain their health; as a result, the proportion of walking or using bicycles during school hours increased. Thus, there was no change in commuting by bicycle or walking to school because adolescents chose to maintain their habitual commuting method, encouraged by the infection prevention education that lasted three years. It can be assumed that these factors contributed to the fact that there was no change in the students’ physical activity when commuting. To enable adolescents to commute to school by bicycle or walking, it is important to create safe roads and bicycle paths. It is also necessary to organize a health campaign that emphasizes that commuting to school by cycling or walking is good for health.

However, this study has some limitations, which suggest areas for future research. First, because the study involved a survey of adolescents in Korea, it is difficult to generalize the results to other countries with different educational and sanitary environments. Future research should reflect and compare the situation in other countries as well. Second, as this study was conducted as quantitative research through a self-reported online survey, it is difficult to include a deep understanding and interpretation of the relationship between COVID-19 and adolescent physical activity participation. Future studies should use qualitative research methods, such as in-depth interviews or mixed research methods, for more in-depth results. Third, the scale used in this study effectively compared the physical activity of adolescents during and after COVID-19. However, it was not possible to comprehensively examine the relationship with other variables (e.g., social relationships, psychological stability, euphoria, and life satisfaction) using the scale. In future, studies to verify the relationship with other variables using IPAQ will be needed.

As suggested in our hypothesis, MVPA was higher after COVID-19 than during COVID-19. These results can be seen as being caused by a decline in school sports and the government’s policies encouraging ‘social distancing’ and ‘uncontact’, which inevitably reduced the physical activity of adolescents during the COVID-19 period. Korean adolescents spend a lot of time preparing for college entrance exams; so, they generally spend most of their lives at school and home. It is difficult to deliberately take time to participate in physical activities; therefore, it is necessary to prepare a plan so that students can take time to participate in physical activities by utilizing school, home, and commuting time.

### 4.2. Practical Implications

The COVID-19 pandemic, which officially began in 2019 and spread globally, has caused major changes in society. Notably, the pandemic has led to the spread of a non-contact culture that requires people to avoid face-to-face contact, with a rapid increase in remote education and telecommuting. In the post-COVID-19 era, the changes caused by COVID-19 have left permanent marks on society that will affect us going forward. Experts predict that a country’s ability to respond to major disasters, e.g., infectious diseases such as the COVID-19 crisis, will be a key criterion for evaluating a country’s capabilities in the evaluation of economic levels and industrial development in developed countries. This is because a country’s response to an infectious disease crisis, including its medical system and civic consciousness, was highlighted during the COVID-19 crisis.

Physical activity in adolescence is linked to lifelong physical activity and health; therefore, it is necessary to have sufficient experience with physical activity in school. However, adolescents who have lived through the pandemic are likely to have difficulty forming these habits if they have not had sufficient physical activity experience at school. Forming correct habits for adolescents to lead healthy lives is an important task for schools; various measures are needed to educate adolescents in this regard.

The implications of the results are as follows. Over the past three years, COVID-19 has created numerous challenges for society and us; it has also made us realize that thorough preparation for a pandemic is necessary. In particular, during the COVID-19 pandemic, people recognized the importance of health and hygiene. As a result, many studies have shown that sufficient physical activity is needed to maintain health [48,49,50]. Pandemics caused by infectious diseases can occur at any time. As such, we should prepare various policies and adjust people’s lifestyles to be ready for them [51]. In particular, schools, where young people spend significant time learning, should implement measures that ensure the correct amount of physical activity for young people, even in the post-COVID-19 era.

This study suggests the following measures to achieve this. First, outdoor activities or light exercise with families should be encouraged. Through this, adolescents can maintain healthy lifestyles and strengthen bonds with family members. Second, schools should organize their curricula so that adolescents can engage in sufficient physical activity. From this, adolescents can develop physical activity habits based on sufficient physical education time at school, encouraging them to experience various physical activities as physical education participants. Third, school sports clubs should be actively encouraged. Many studies have reported the benefits of school sports club activities, such as proper exercise methods and techniques that can be learned from professional instructors [52]. Furthermore, participating in school sports club activities enables adolescents to enjoy sports and social exchanges with friends [53,54]. Thus, adolescent educational institutions should encourage physical activity in school sports clubs so that adolescents acquire physical and social health benefits. Fourth, adolescent educational institutions need to promote an environment where physical activities can become habitual in daily life. As the results show, when residences are not too far from the school, students can commute by walking or cycling. Overall, there is a need to make light physical activities a habit and to create an environment where people can be active, such as by using stairs rather than elevators. To ensure sufficient physical activity for adolescents, adolescent educational institutions need to encourage various efforts in communities, schools, and homes.

The COVID-19 pandemic has caused both unprecedented disruptions and massive changes in education [55]. However, as humanity emerges from the pandemic, it is trying to get back to its former routine. While the pandemic has caused a lot of damage, it has also delivered lessons such as the importance of a ‘hygienic living environment and health’. Various studies [56,57,58] have predicted that there will be changes in various aspects of life after COVID-19. In particular, as argued by Serafin-Munñoz et al. [59], it is necessary to make efforts from various angles to prevent new pandemics and to maintain healthy lifestyles for adolescents beyond the three pillars of the environment, economy, and society.

However, this study has some limitations, which suggest areas for future research. First, the main purpose of this study was to compare the differences in physical activities of adolescents during and after COVID-19. However, despite investigating the economic levels at home and the academic performances of the adolescents in this study, the difference in physical activity was not verified with regard to the main purpose and the scope of verification of the study. Therefore, in subsequent studies, a study will be needed to verify the difference in physical activity of adolescents according to the economic level and academic achievement level of the adolescents. Second, because this study conducted a survey of adolescents in Korea, it is difficult to generalize the results to other countries with different educational and sanitary environments. Future research should reflect and compare the situation in other countries as well. Third, since this study conducted this investigation as quantitative research through a self-reported online survey, it is difficult to include a deep understanding and interpretation of the relationship between COVID-19 and adolescent physical activity participation. Future studies should use qualitative research methods, such as in-depth interviews or mixed research methods, for more in-depth results. Fourth, the scale this study used effectively compared the physical activity of adolescents during and after COVID-19. However, it was not possible to comprehensively examine the relationship with other variables (e.g., social relationships, psychological stability, euphoria, life satisfaction, etc.) using the scale. In future studies, methods to verify the relationship with other variables using IPAQ will be needed.

## 5. Conclusions

This study aimed to verify differences in adolescent physical activity during and after COVID-19. To achieve this, using online Google Forms with an IPAQ long version questionnaire, 1143 Korean adolescents were surveyed in 2022 and 2023. The findings indicated that high-, moderate-, and low-intensity physical activities and total leisure-time physical activities were significantly higher after COVID-19 than during the pandemic. Additionally, high-, moderate-, and low-intensity physical activities and total physical activities in schools were significantly higher after COVID-19 than during it. There was no statistically significant difference in physical activity during school commute time, for either cycling or walking, or in the total physical commuting activity during and after COVID-19.

In the context of the COVID-19 pandemic, the decrease in physical activity among adolescents has created many challenges in the field of physical education. In the post-COVID-19 era, physical education teachers have been working to encourage adolescent physical activity as a top priority in school physical education to lay the foundation for healthy lifestyles. Considering the implications of this study’s results and the measures proposed, efforts would be needed to apply them in schools, along with follow-up studies that track their effectiveness going forward.

## Figures and Tables

**Table 1:** General characteristics of the participants.

Characteristics	Categories	During COVID-19 (*n* = 570)		After COVID-19 (*n* = 573)	
		*n*	%	*n*	%
Sex	Male	264	46.3	313	54.6
	Female	306	53.7	260	45.4
Economic level in house	High	117	20.6	127	22.2
	Middle	397	69.6	396	69.1
	Low	56	9.8	50	8.7
Academic performance	High	84	14.8	136	29.6
	Middle	271	47.5	272	47.5
	Low	115	37.7	165	28.8
PAPS (Physical Activity Promotion System) grades	Grade 1	110	19.3	179	31.2
	Grade 2	169	29.6	165	28.8
	Grade 3	203	35.6	175	30.5
	Grade 4	58	10.2	39	6.8
	Grade 5	30	5.3	15	2.6
Obesity level	Underweight	198	34.7	226	39.4
	Normal	275	48.3	249	43.5
	Mild obesity	74	13.0	73	12.7
	Obesity	23	4.0	25	4.4
Total		570	100.0	573	100.0

Tested by frequency analysis. Physical Activity Promoting System (PAPS) is a comprehensive physical fitness evaluation system that is mandatorily implemented in elementary, middle, and high schools under the School Sports Promotion Act in Korea. PAPS is classified into grades 1 to 5 (1st grade = very good, 5th grade = highly insufficient) [36].

**Table 2:** Reliability of International Physical Activity Questionnaire.

Variables	Cronbach’s α
Leisure-time physical activities	0.845
Physical activities in school	0.838
Physical activities during commute	0.773
Moderate-to-vigorous physical activity	0.730

Tested by internal consistency reliability analysis.

**Table 3:** Differences in moderate-to-vigorous physical activity during and after COVID-19.

Variable (Minutes/Week)	During COVID-19	After COVID-19	t	*p*	Effect Size (d)	95% Confidence Interval of the Difference	
						Lower Limit	Upper Limit
Moderate-to-vigorous physical activity	337.78 ± 837.95	450.73 ± 778.85	−2.360	0.018 *	0.14	−206.86	−19.05

Data are expressed as mean ± standard deviation. * p < 0.05, tested by independent *t*-test.

**Table 4:** The differences in leisure-time physical activities during and after COVID-19.

Variable (Minutes/Week)	During COVID-19	After COVID-19	t	*p*	Effect Size (d)	95% Confidence Interval of the Difference	
						Lower Limit	Upper Limit
High-intensity leisure-time physical activity	161.42 ± 423.54	221.51 ± 435.95	−2.363	0.018 *	0.14	−109.97	−10.20
Moderate-intensity leisure-time physical activity	176.36 ± 429.18	229.23 ± 395.01	−2.167	0.030 *	0.13	−100.75	−4.99
Low-intensity leisure-time physical activity	326.67 ± 317.04	395.42 ± 432.98	−3.064	0.002 **	0.18	−112.78	−24.72
Total leisure-time physical activity	664.45 ± 986.05	846.15 ± 1078.58	−2.973	0.003 **	0.18	−301.63	−61.78

Data are expressed as mean ± standard deviation. * *p* < 0.05 ** *p* < 0.01, tested by independent *t*-test.

**Table 5:** The differences in physical activities in school during and after COVID-19.

Variable (Minutes/Week)	During COVID-19	After COVID-19	t	*p*	Effect Size (d)	95% Confidence Interval of the Difference	
						Lower Limit	Upper Limit
High-intensity physical activities in school	53.55 ± 170.44	85.45 ± 212.97	−2.797	0.005 **	0.17	−54.29	−9.52
Moderate-intensity physical activities in school	49.89 ± 157.48	80.31 ± 190.97	−2.939	0.003 **	0.35	−50.73	−10.11
Low-intensity physical activities in school	73.70 ± 104.62	97.00 ± 154.43	−2.988	0.003 **	0.18	−38.61	−8.00
Total physical activities in school	177.14 ± 364.32	262.76 ± 464.95	−3.466	0.001 **	0.20	−134.09	−37.16

Data are expressed as mean ± standard deviation. ** *p* < 0.01, tested by independent *t*-test.

**Table 6:** Differences in physical activities in commute time during and after COVID-19.

Variable (Minutes/Week)	During COVID-19	After COVID-19	t	*p*	Effect Size (d)	95% Confidence Interval of the Difference	
						Lower Limit	Upper Limit
Commute time by bicycle	22.04 ± 85.94	25.14 ± 74.85	−0.651	0.515	0.04	−12.45	6.25
Commute time by walking	134.29 ± 171.20	141.05 ± 154.67	−0.700	0.484	0.04	−25.69	12.18
Total physical activities in commute time	156.32 ± 166.18	166.18 ± 180.64	−0.888	0.375	0.06	−31.65	11.94

Data are expressed as mean ± standard deviations. Tested by independent *t*-test.

## Data Availability

The original contributions presented in the study are included in the article, further inquiries can be directed to the corresponding author.
